# Impact of a semi-structured briefing on the management of adverse events in anesthesiology: a randomized pilot study

**DOI:** 10.1186/s12871-019-0913-5

**Published:** 2019-12-18

**Authors:** Christopher Neuhaus, Johannes Schäfer, Markus A. Weigand, Christoph Lichtenstern

**Affiliations:** Department of Anesthesiology, Heidelberg University Hospital, Im Neuenheimer Feld, 110 69120 Heidelberg, Germany

**Keywords:** Human factors, Briefings, Checklists, Mental models, Airway management, Simulation

## Abstract

**Background:**

Human factors research has identified mental models as a key component for the effective sharing and organization of knowledge. The challenge lies in the development and application of tools that help team members to arrive at a shared understanding of a situation. The aim of this study was to assess the influence of a semi-structured briefing on the management of a simulated airway emergency.

**Methods:**

37 interprofessional teams were asked to perform a simulated rapid-sequence induction in the simulator. Teams were presented with a “cannot ventilate, cannot oxygenate” scenario that ultimately required a cricothyroidotomy. Study group (SG) teams were asked to perform a briefing prior to induction, while controls (CG) were asked to perform their usual routine.

**Results:**

We observed no difference in the mean time until cricothyroidotomy (SG 8:31 CG 8:16, *p* = 0.36). There was a significant difference in groups’ choice of alternative means of oxygenation: While SG teams primarily chose supraglottic airway devices, controls initially reverted to mask ventilation (*p* = 0.005). SG teams spent significantly less time with this alternative airway device and were quicker to advance in the airway algorithm.

**Conclusions:**

Our study addresses effects on team coordination through a shared mental model as effected by a briefing prior to anesthesia induction. We found measurable improvements in airway management during those stages of the difficult airway algorithm explicitly discussed in the briefing. For those, time spent was shorter and participants were quicker to advance in the airway algorithm.

## Background

Over the last decade, the importance of effective interprofessional teamwork in healthcare has emerged as one of the main factors behind the safe provision of care. While the exact definition of “effective” remains unclear, a variety of models and frameworks have tried to approximate and operationalize teamwork and identify underlying core concepts and principles [1, 2]. Among those, human factors research across a variety of high-consequence industries has identified team mental models (TMMs) as one of the key components for the effective sharing and organization of knowledge [3–5]. They have to be understood as internal representations of a complex system that allow an individual to interact with the system and understand its behavior, dynamics and performance [6]. The development and sharing of team mental models, more commonly known as “being on the same page”, has repeatedly demonstrated positive effects on team performance [7]. In theory, a shared TMM helps team members in anticipating each other’s actions and facilitates coordination especially in dynamic, stressful situations where opportunities for communication are limited [3, 5]. The practical challenge lies in the development and application of tools that help team members with aligning different mental models to arrive at a shared understanding of an upcoming situation. One solution lies in the form of briefings [8, 9], or short and focused, semi-structured opportunities for information exchange. The aim of this study was to assess the influence of a semi-structured briefing on the management of a simulated airway emergency in anesthesiology.

## Methods

### Research ethics

This study was approved by the Ethics Committee of the Medical Faculty, University of Heidelberg (S-521/2015). Written informed consent was obtained from all participants. This manuscript adheres to the applicable EQUATOR guidelines.

### Study design

37 interprofessional teams consisting of one anesthetist and one anesthesia nurse from a large university hospital volunteered for this study. They were asked to perform a simulated rapid-sequence induction (RSI) in the simulator (Human Patient Simulator HPS, CAE Healthcare, Sarasota, FL, USA). Teams were assigned to either study group (SG) or control group (CG) using stratified randomization (tiers were board-certified vs. trainee). In the ensuing scenario, all teams were presented with a “cannot ventilate, cannot oxygenate” (CVCO) scenario that ultimately required a cricothyroidotomy. Study group teams were asked to perform a briefing prior to the induction, while controls were asked to perform their usual routine. All participants were familiar with the simulation environment due to regular departmental simulation training; however, before starting the study they were introduced to the simulator and could familiarize themselves with the equipment and surroundings. The study commenced only after any open questions were answered by the investigators. Participants were blinded to the study hypothesis and primary outcome measure. They did not receive compensation for their participation.

### TEAM briefing

**Fig. 1:**
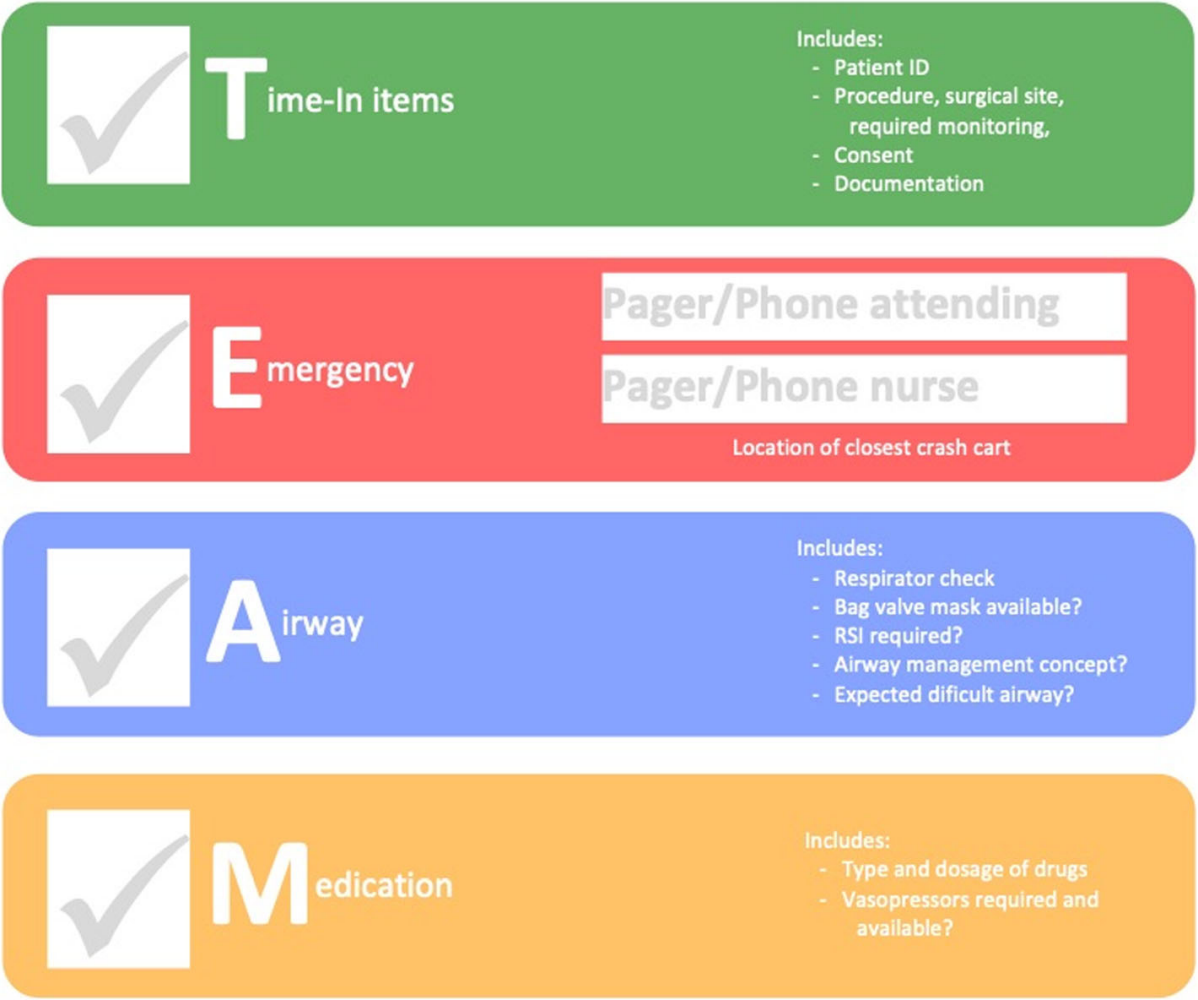
TEAM framework as published in [9]

We previously published the mnemonic TEAM to provide a framework (Fig. 1) for semi-structured briefings in anesthesia [9]:

- Time-in items: Stress any findings from the sign-in checklist relevant to patient safety.
- Emergency: In case of a problem during the induction of anesthesia, available personnel and equipment and their location shall be known. This includes pager/phone numbers of physicians and nurses in supervisory roles and the location of the nearest crash/airway cart.
- Airway: A strategy for securing the patient’s airway, including the risk assessment for aspiration and difficult airway management options, should be discussed, and the required equipment needs to be verified available and checked.
- Medication: The planned type of anesthesia should be discussed, including the type and estimated dosage of drugs. The requirement for additional drugs readily available at the time of induction depending on pre-existing medical conditions should be considered (e.g., vasopressors for patients with cardiac conditions).

Members of the study group watched a 7-min instructional video on the purpose and execution of a briefing using the TEAM framework, and instructors were available to clarify any remaining questions or uncertainties. None of the participants had prior training or experience in the TEAM mnemonic.

### Case

In the simulator, teams were confronted with a 22-year-old male patient presenting with acute appendicitis. Two minutes after the induction (as defined by the application of first opioid or hypnotic medication), the patient started to desaturate according to the underlying physiological model (“Standard man”, METI HPS6, CAE Healthcare, Sarasota, FL, USA). Primary endpoint was the decision to perform a cricothyroidotomy. Secondary endpoints were the timing and methods used in airway management and the timing of calling for help.

### Statistical analysis

Data was analyzed descriptively with absolute and relative values and their mean values and standard deviation. For the primary and secondary endpoint, time differences between groups were compared using a log-rank test stratified for experience. Influences of participant experience on timing were assessed using Cox-regression. Hazard ratios were determined together with 95% confidence intervals. For the secondary endpoints in regard to methods used in airway management and adherence to existing guidelines, Mann-Whitney-U test and Chi [2]-test were used to compare continuous and categorial data, respectively. A *p*-value < 0.05 was considered statistically significant. These have a purely descriptive character, need to be interpreted accordingly and possess no confirmatory value. Missing values were not imputed. As this was an exploratory pilot trial, no power calculation could be conducted in the planning phase. The sample size was instead based on considerations of feasibility.

## Results

Of the 37 teams participating in the study, 19 were randomly assigned to perform briefings in the study group, while 18 teams remained in the control group. Demographic data is presented in Table 1.

**Table 1:** Participants’ demographics

	Anesthetists		Nurses		
	Work experience (years)	# of inductions	Work experience (years)	# of inductions	
Control group					
Median	4,00	2000,00	15,00	600,00	
IQR	3,0	1350,00	17,5	4350,00	
Study group					
Median	4,00	1800,00	10,00	1200,00	
IQR	5,0	2700,00	17,0	3700,00	
p	0.768	0.249	0.704	0.881	
	Age				
	18–25	26–35	36–45	46–55	> 55
Control group					
Anesthetists	0	10 (58,8%)	7 (41,2%)	0	0
Nurses	0	8 (47,1%)	6 (35,3%)	2 (11,8%)	1 (5,9%)
Study Group					
Anesthetists	0	14 (73,7%)	5 (26,3%)	0	0
Nurses	2 (10,5%)	6 (31,6%)	5 (26,3%)	5 (26,3%)	1 (5,3%)

**Fig. 2:**
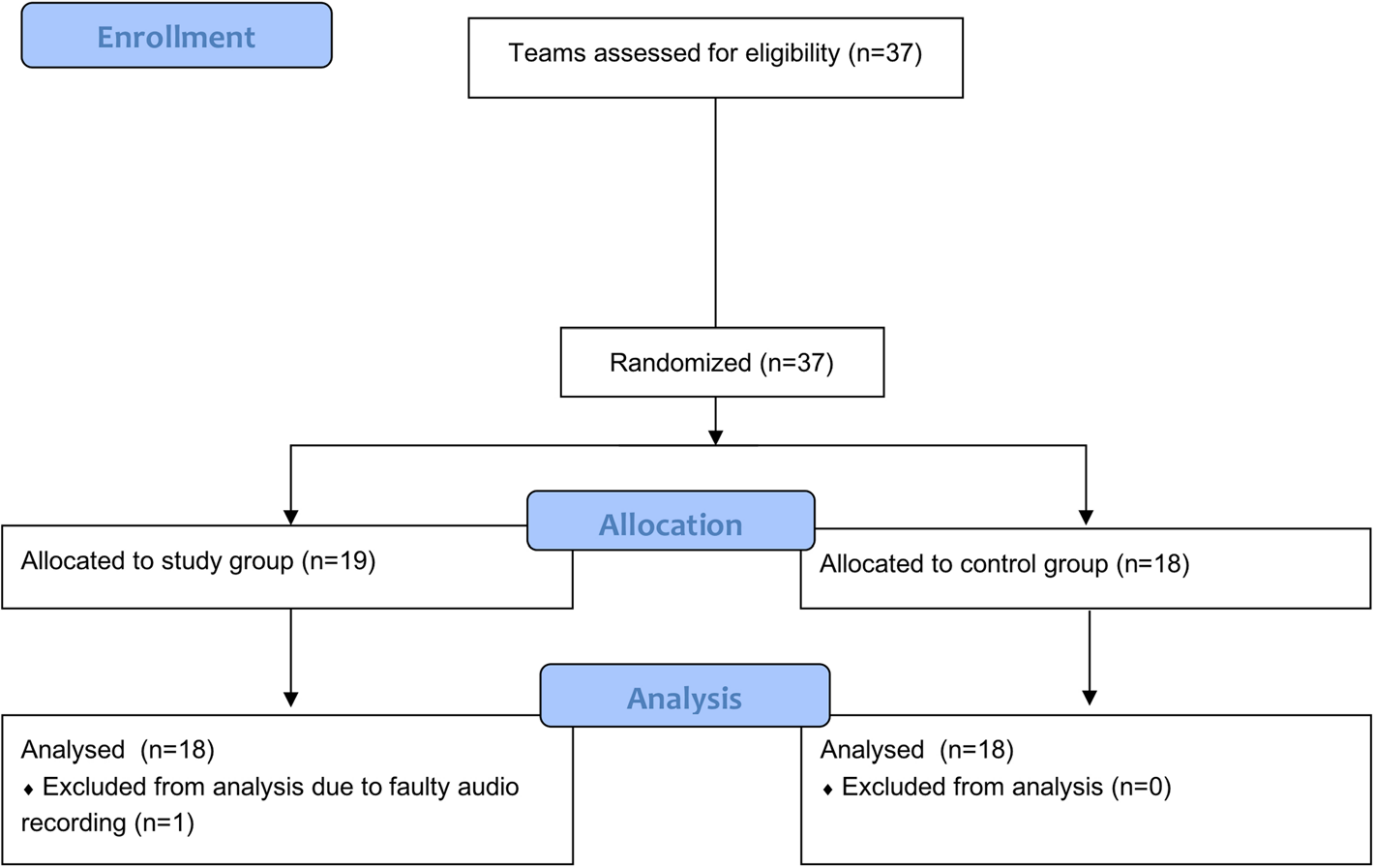
CONSORT Flow Diagram

**Fig. 3:**
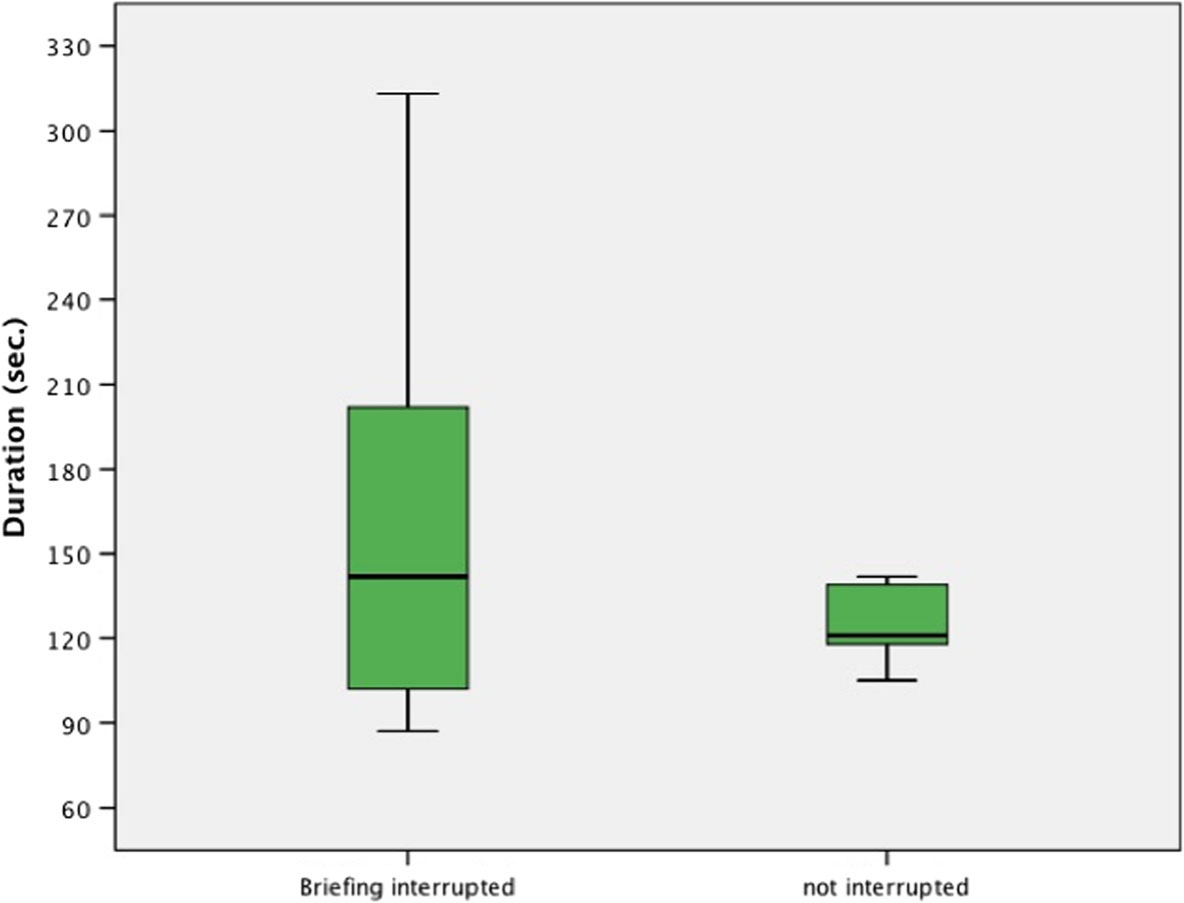
Average briefing duration

Due to a faulty audio recording, data from one team in the control group could not be analyzed (see Fig. 2). Briefings in the study group had an average duration of 2:28 min (SD 60s, Fig. 3). 11 teams chose to interrupt the briefing to immediately perform tasks that had just been discussed (e.g. the preparation of vasoactive medication, verifying the availability of a laryngeal mask as alternative airway, insertion of a gastric tube) before resuming the TEAM briefing. This prolonged the briefing for an average of 36 s but had no significant impact on the primary endpoint (*p* = 0.44). In the study group, 42% of teams (*n* = 8) discussed a primary strategy for alternative airway management (Plan B), while 11% (*n* = 2) discussed an additional secondary one (Plan C). 63% of SG teams (*n* = 12) pre-emptively discussed vasoactive medication, and 42% (*n* = 8) reviewed available emergency equipment. None of the SG teams discussed a cricothyroidotomy (Plan D). In the control group, the observed routine before induction included isolated random exchanges of information (e.g. the desired medication, or ET tube size), but no structured or comprehensive briefing was observed. A comparison of conversational content between groups is provided in Table 2. Notably, we observed significant differences in the discussion of available emergency equipment (*p* = 0.002) and contact information in the case that help should be required (*p* = 0.047).

**Table 2:** Comparison of relevant briefing content covered in team conversations

	Airway management strategy	Primary strategy for alternative airway mgmt.	Secondary strategy for alternative airway mgmt.	Vasoactive medication	Emergency equipment	Call for help
Control group	17 (100%)	3 (17,7%)	0 (0%)	10 (52,9%)	0 (0%)	6 (35,4%)
Study Group	19 (100%)	8 (42,1%)	2 (10,5%)	12 (63,2%)	8 (42,1%)	13 (68,4%)
p	0.056	0.112	0.169	0.535	0.002	0.047

**Fig. 4:**
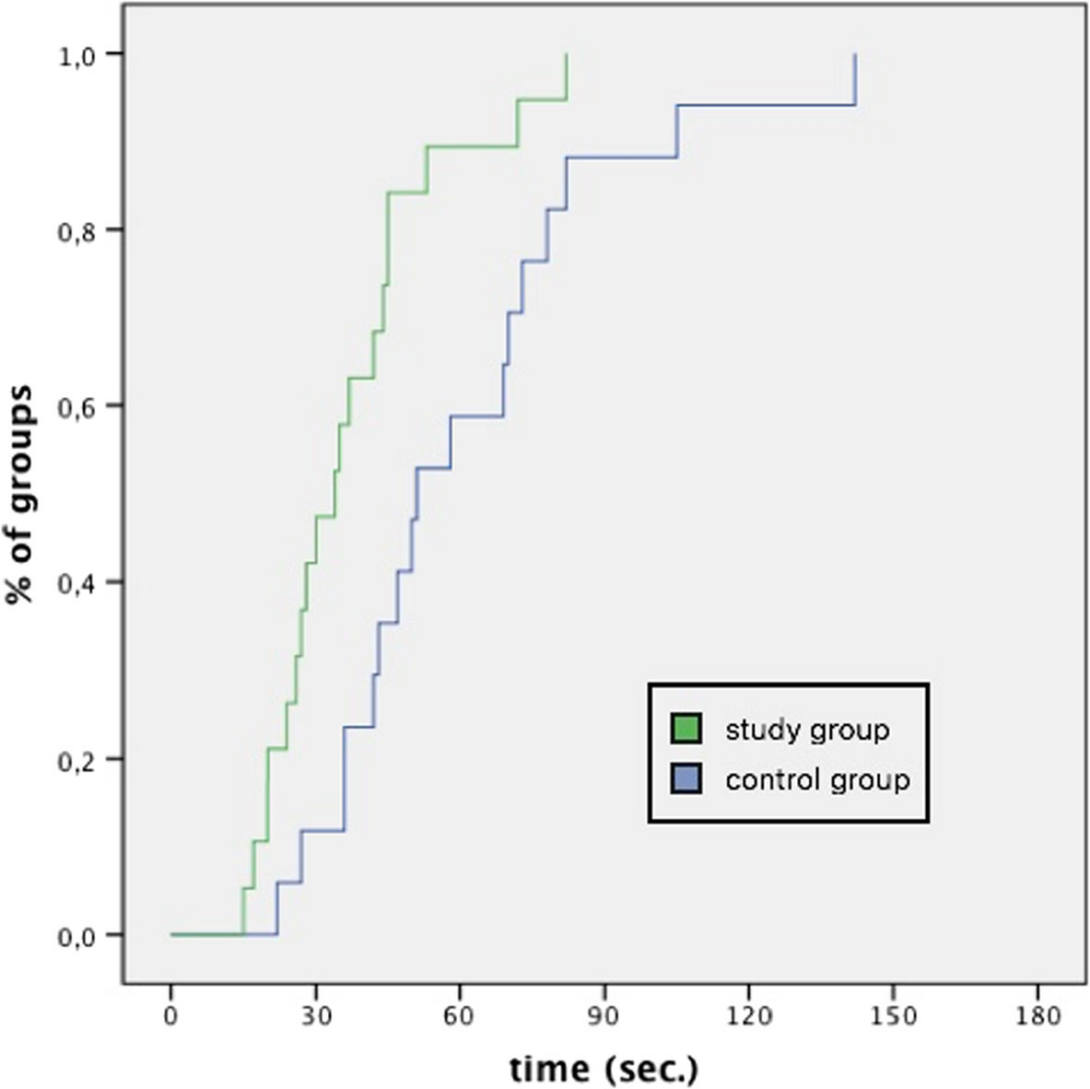
Time used for airway management using the first alternative airway device

During the scenario, we observed no significant difference between groups regarding the timing for switching to the first alternative airway device (Plan B) after failed endotracheal intubation. There was a significant difference between groups in their choice of alternative means of oxygenation: While teams in the study group primarily chose supraglottic airway devices, controls initially reverted to mask ventilation (*p* = 0.005). Moreover, teams in the study group (SG) spent significantly less time with this alternative airway device than controls (CG) and were quicker to advance in the airway algorithm towards Plan C (Fig. 4). We observed no difference in the mean time until mentioning (SG 6:27 min, CG 6:49 min, *p* = 0.63) or performing a cricothyroidotomy (Plan D; SG 8:31 CG 8:16, *p* = 0.36). However, the elapsed time until a decision to perform a cricothyroidotomy was made significantly correlated with the experience of the anesthesiologist in all participating groups (*p* = 0.019, 95% HR 1.109, CI 1.017–1.209).

A significant larger number of teams (*n* = 13, 68%) in the study group explicitly mentioned the emergency contact number during the briefing, compared to *n* = 6 (35%) in the control group. Throughout the scenario, we observed no significant difference between groups in the timing of the call for help. Ultimately, the mere mention of contact information had no impact on how early a call for help was placed (*p* = 0.32).

## Discussion

Together with increasing awareness for patient safety in general, the seminal Institute of Medicine report “To Err Is Human” [10] was one of the first publications that highlighted the importance of team performance in healthcare and inspired subsequent research. One of the predominant definitions of a team is “a set of two or more individuals interacting adaptively, interdependently and dynamically towards a common and valued goal” [11]. Manser [12] further highlights aspects that are especially relevant for healthcare, among them task-specific competencies and specialized work roles while using shared resources. In anesthesiology, due to the domain’s dynamic nature and coupled with the fact that teams have changing membership and are often assembled “ad-hoc”, this reinforces the need for high quality coordination and communication [12, 13]. In this context, the concept of shared team mental models (TMM) is used to describe complex human interaction that includes anticipating each other’s actions, simplifying coordination and improving collaboration [3, 5]. The present study explores the application of a semi-structured briefing as one possible tool often used for the alignment of TMMs in various high-consequence industries to anesthesiology.

Contrary to our hypothesis, our study showed no significant difference between groups in the time spent on the decision to perform an emergency cricothyroidotomy. This may be due to several reasons. It has to be stressed that none of the SG teams explicitly discussed this procedure during the briefing. For those parts of the airway algorithm that participants chose to discuss, usually a supraglottic airway device as first alternative (Plan B) and mask ventilation as second alternative (Plan C), we noted a significant difference between groups in the time spent with those alternatives and in the advancement in the algorithm. However, this effect did not implicitly “spill over” to the rest of the airway algorithm. These findings further add to contradictory results on the impact of structured mental rehearsal of activity on subsequent performance: A study by Hayter et al. demonstrated that a structured mental practice did not lead to any difference in observed nontechnical skills and no difference in time to perform chest compressions, administer epinephrine, and give blood in a simulated cardiac arrest [14]. However, Lorello et al. demonstrated significantly improved teamwork according to a validated team-based behavioral rating scale after structured mental rehearsal [15].

Emergency cricothyroidotomies remain rare events (approximately 1:50.000 anesthetics) that anesthesiologists do not necessarily feel comfortable or experienced with, and that are not trained on a regular basis [16]. Skill retention rates for cricothyroidotomies have been shown to range between 3 to 6 months and 1 year, depending on the technique [17]. The ensuing doubts and hesitation associated with an invasive, unfamiliar and potentially risky procedure are apparently not overcome by a semi-structured pre-induction briefing that discusses various contingencies, but that is primarily designed for the individually adaptive alignment of mental models and not specifically for review of complete difficult airway guidelines. In that context, it is especially interesting to note the significant influence of anesthesiologists’ experience on the decision to perform a cricothyroidotomy. Taken together. Our study reinforces the need for regular training in airway management, including percutaneous emergency cricothyroidotomy. It has been repeatedly shown that a combination of delayed decision making, skill deficits and inappropriate knowledge impedes timely execution of emergency front-of-neck access in CVCO situations [17, 18]. From a human factors perspective, it remains to be studied how the decision making is influenced by the latter two factors. In that regard, it is debatable if a CVCO scenario is ideally suited to demonstrate the benefits of a TEAM briefing, as it is neither very ambiguous nor very complex, but subject to confounding difficulties not overcome by our intervention.

One of the key findings of this study is that a team briefing in anesthesiology that is adaptively focused on the management of certain contingencies *can* significantly improve the efficiency of the ensuing actions, provided that these aspects are explicitly discussed during the briefing. In our example, after failed endotracheal intubation, while SG teams primarily reverted to a supraglottic airway device and quickly moved on after realizing that this alternative did also not lead to sufficient oxygenation (as discussed in their briefing), CG teams initially reverted to mask ventilation while discussing and coordinating the teams’ next move. Consequently, the investment of a few minutes before induction that included discussion of initial alternative airway strategies lead to a smoother, more focused initial approach to airway management in a simulated airway emergency, since most necessary team coordination had already taken place during the briefing. This could potentially save precious seconds in a real-life situation where the patient cannot be oxygenated.

While guidelines provide a good frame of reference for a certain situation, the exact course of action is still dependent on individual decisions that need to be communicated within the team. The explicit communication in form of instructions or orders commonly used to coordinate the team has been shown to be impaired in dynamic, stressful situations [19]. Successful joint activity is dependent on interpredictability and “common ground”, or “pertinent knowledge, beliefs and assumptions that are shared among the involved parties” [20]. Through anticipation and deliberate, proactive communication strategies, teams with shared mental models have been shown to work faster and more effectively. This implicit form of coordination can help to facilitate team interaction [21].

In this regard, it is important to reinforce the difference between semi-structured briefings and checklists, as we have previously done [9]. This differentiation is largely unknown in medicine, where the term checklist is used synonymously for a multitude of tools used to promote procedural standardization and increase patient safety. Other domains, like aviation, clearly distinguish between, teach and apply briefings and checklists at different stages during a flight in an effort to harness the positive effects of combining multiple tools [9]. In theory, checklists, which have also been proposed as a pre-induction measure to improve safety [22], are used to verify critical steps in a procedural workflow. They are especially well suited for standardized work that has minimal to no variation. On the other hand, briefings are a more informal addition that serve a multitude of purposes. They help with the alignment of mental models within the team, while facilitating, or “opening up”, communication [4, 23]. But more importantly, briefings introduce an element of adaptability that complements the rigid content found in checklists. They help to harness the adaptive capacity of humans collaborating towards a common goal by providing an opportunity to highlight special considerations in a given situation or case, direct attention and focus on peculiarities and exceptions to the usual routine. By doing so, they foster a more resilient style of work that can help advance patient safety efforts from the traditional, reactive focus on “fixing things that went wrong” to a more proactive, vigilant state where things “keep on going right” [24]. Briefings support the incorporation of properties such as education, training, experience or intuition into applied patient safety in a collective rather than merely individual fashion.

In the current study, increased work efficiency and quicker decision making were observed in the areas covered by the briefing, usually the first and sometimes second alternative approach to airway management. This was achieved with an investment in training of around 10 min that could be considered minimal, further hinting at the potential benefit of briefings when implemented on a larger, more robust scale. The exchange of information that could be observed in the control group, while mostly unstructured, shows that communication and collaboration are central, intuitive components of teamwork. However, in current anesthesia practice heavily focused on proceduralised (read checklist) work, this remains unsupported and is left to be taken care of by individual chance. The TEAM-framework/mnemonic can serve to structure pre-induction communication while at the same time providing a measure of focus on certain aspects that are generally considered important for anesthetic practice.

To date, there is no scientific method to devise mnemonics other than expert opinion, “trial and error” and comparative studies. As previously published debates (e.g. about FAST-HUG [25] in intensive carae) have shown, the challenge lies in finding a mnemonic that is poignant and short enough to be readily remembered and applied in practice, but not too generic or broad to be of little value to the clinician [26, 27]. The areas covered by TEAM can, and should, be regularly assessed for their ability to strike this balance and reflect critical areas of perioperative patient safety, and be modified if the need arises.

Particularly interesting is the lack of difference between groups regarding the call for help. Considering how the provision of anesthesia is generally organized, managing and optimizing resources could be considered a key feature in managing adverse events, in marked contrast to industries traditionally associated with briefings (e.g. aviation) where additional help is rarely available. Although a significantly higher number of teams in the study group explicitly reviewed emergency contact information, this did not result in an earlier call for help. One possible explanation is that in certain departmental cultures, help is called as the result of running out of options or a perceived loss of control rather than in an effort to utilize all available resources. In this regard, briefings could potentially further delay an early call for help by scripting and organizing actions for a team, thereby giving team members an increased sense of control. Special care needs to be taken when implementing and training the use of briefings to emphasize the benefit that can be harnessed from an early call for help.

Concerning the potential implementation of briefings into anesthesia practice, our study can help objectify often-raised concerns about “hidden” costs of introducing human factors tools in the OR because of the time that is spent. Our data shows that a briefing can be performed in a very short amount of time. While finding suitable metrics for cost-benefit discussions of briefings will be next to impossible using traditional quantitative measurements, the relatively short duration of briefings demonstrated this study might help alleviate some of the concerns attached to process optimization in the OR environment.

Our study has several limitations. First and foremost, as this was a simulator study, there is always the expectancy bias that an adverse event is about to occur. As participants were observed outside of their normal work environment and routine, one has to be cautious with the interpretation of behavior in relation to real-life situations. This simulator bias might have had a significant effect on the decisions to perform a cricothyroidotomy, and when to call for help.

Second, the training and familiarization time with the TEAM-briefing tool was relatively short. While our results showed promising effects, after the video explanation a disappointingly small number of study group teams discussed alternative airway management despite this being the A in TEAM. Semi-structured briefings are designed with ample leeway for individual interpretation; however, a modified instructional strategy might help teams follow the TEAM tool more closely. A more thorough implementation might help improve teamwork significantly through a more complete alignment of TMMs. It has to be noted, however, that actions and behaviors do not necessarily equate with understanding of the situation.

Third, our study was an exploratory pilot trial, hence, no power calculation could be conducted in the planning phase. The sample size was instead based on considerations of feasibility. Consequently, our trial might not have been adequately powered to detect differences between treatment groups. This is especially true if the dynamic nature of the scenario is considered, where treatment times between groups remain close, therefore requiring a large sample size.

Fourth, due to the study design, we singularly focused on a difficult airway scenario, and evaluated the briefing effects accordingly. This approach does not necessarily represent or capture the diverse and complex web of human interactions taking place in a dynamic work environment. The primary endpoint for this study, while ideally suited for a quantitative analysis, might not be optimally chosen to demonstrate the benefits of a briefing. A more ethnographical approach might be better suited to evaluate the intricate subtleties found in multi-professional teamwork, and could further our understanding of the complex process that is human everyday work.

While our study showed mixed results in the areas affected by the briefing, we had no indication that communication, collaboration and crisis management were impaired, or worsened, in the study group. Consequently, the results of this study warrant a larger follow-up investigation into the effects of anesthesiologic briefings in an actual work environment. Of special interest are questions regarding the effectiveness in regard to the amount of proceduralization of a certain tool. It is unclear whether “interrupting” a briefing negatively impacts the briefing message, concentration/focus, and ultimately generation of a shared mental model within the team. This aspect is not addressed in its entirety by our study, since our primary endpoint didn’t necessarily reflect the shared cognitive workload within a team.

## Conclusion

Our study addresses effects on implicit team coordination through a shared team mental model as effected by a team briefing prior to anesthesia induction. We found measurable improvements in airway management during those items of the difficult airway algorithm explicitly discussed in the briefing. For those, time spent was shorter and participants were quicker to advance in the airway algorithm in a simulated “cannot ventilate, cannot oxygenate” scenario. Further studies are warranted to explore the influence of briefings as tools for increased patient safety in the OR.

## Data Availability

The datasets used and/or analysed during the current study are available from the corresponding author on reasonable request.

## Abbreviations

CG: Control group
CVCO: Cannot ventilate, cannot oxygenate
OR: Operating room
RSI: Rapid sequence induction
SD: Standard deviation
SG: Study group
TMM: Team mental models
